# Primary Ciliary Dyskinesia: Ancestral Haplotypes Analysis of the RSPH4A Founder Mutation in Puerto Rico

**DOI:** 10.7759/cureus.17673

**Published:** 2021-09-03

**Authors:** Wilfredo De Jesús-Rojas, Dalilah Reyes De Jesús, Angélica M Nieves, Ricardo A Mosquera, Juan C Martinez-Cruzado

**Affiliations:** 1 Department of Pediatrics, University of Puerto Rico, Medical Sciences Campus, San Juan, PRI; 2 Department of Pediatrics, Ponce Health Sciences University, Ponce, PRI; 3 Department of Pediatrics, San Juan Bautista Medical School, Caguas, PRI; 4 School of Medicine, Ponce Health Sciences University, Ponce, PRI; 5 Division of Pediatric Pulmonology, University of Texas (UT) Physicians High Risk Children's Clinic at McGovern Medical School at UTHealth, Houston, Texas, USA; 6 Department of Biology, University of Puerto Rico in Mayagüez, Mayagüez, PRI

**Keywords:** primary ciliary dyskinesia, rsph4a, ancestry, founder mutation, puerto rico

## Abstract

Genetic mutations in >50 genes, including *RSPH4A,* can lead to primary ciliary dyskinesia (PCD). *RSPH4A* mutations affect radial spokes, which alter the configuration of the ciliary ultrastructure and lead to chronic oto-sinopulmonary disease. The *RSPH4A* [c.921+3_6delAAGT] founder mutation was described as one cause of PCD without laterality defects in Puerto Rico. The average Puerto Rican genetic composition includes 64% European, 21% African ancestral, and 15% Native-American or Taino, a native tribe in the Caribbean at the start of the European colonization, genes. Due to the relatively elevated Taino ancestry on the island, it might have contributed to the endemicity of the *RSPH4A* [c.921+3_6delAAGT] splice site mutation. However, the ancestry of this mutation is still not confirmed. This article describes the two pediatric PCD cases with the Puerto Rican foundermutationand reports an ancestral haplotype analysis of the *RSPH4A* [c.921+3_6delAAGT] splice site mutation. A median-joining haplotype network was constructed with the genome sequence data from 104 Puerto Rican subjects in the 1000 Genomes Project (1000GP). This study found that the *RSPH4A* [c.921+3_6delAAGT] splice site mutation was carried to Puerto Rico from Europe by conquistadors or shortly after the conquest and that it gained frequency on the island through genetic drift fueled by a subsequent population expansion.

## Introduction

Primary ciliary dyskinesia (PCD) is a genetically heterogeneous autosomal recessive disorder characterized by motile cilia dysfunction, which affects approximately one in 15,000 individuals in the United States [1]. Genetic mutations in >50 human genes can lead to PCD, affecting the function of many ciliary structural proteins [2-3]. These abnormalities result phenotypically in neonatal respiratory distress in approximately 80% despite a full-term gestation and chronic oto-sinopulmonary disease since birth, as well as male infertility and organ laterality identified later in life in 50% of cases [4]. New guidelines for the diagnosis of PCD have included genetic testing as part of the diagnostic algorithm [5-6]. Several PCD mutations have been discovered as genetic testing has become more accessible to physicians, including mutations affecting the *RSPH4A* gene [7]. *RSPH4A* mutations located in chromosome six affect cilia radial spokes, which alters the configuration and function of the ciliary ultrastructure [8-9]. Previous studies have described several cases of PCD without laterality defects in individuals with Puerto Rican heritage and native Puerto Ricans due to the *RSPH4A* [c.921+3_6delAAGT] (rs869320683) splice site mutation [7,10].

The average Puerto Rican genetic composition includes 64% European, 21% African, and 15% Native-American or Taino ancestral genes [11]. Due to the endemicity of the mutation and the relatively elevated Taino ancestry on the island, *RSPH4A* [c.921+3_6delAAGT] splice site mutation has been described as likely being of Taino origin, the main Native American tribe in the Caribbean at the start of the European colonization [7,12]. However, no previous studies have been completed to explore the ancestry of the *RSPH4A* [c.921+3_6delAAGT] splice site mutation. We present a compound heterozygous and a homozygous pediatric case for the Puerto Rican founder *RSPH4A* [c.921+3_6delAAGT] splice site mutation. Also, we report the ancestral haplotype analysis for the Puerto Rican founder mutation.

Preliminary data of this article were previously presented as a meeting abstract at the 2020 American Thoracic Society Annual Scientific Meeting Am J Respir Crit Care Med 2020;201:A5328. Internet address: https://www.atsjournals.org/doi/pdf/10.1164/ajrccm-conference.2020.201.1_MeetingAbstracts.A5328

## Case presentation

Case 1: *RSPH4A* compound heterozygous male pediatric case

This was a case of an 11-year-old native Puerto Rican male with a past medical history of neonatal respiratory distress despite term gestation, daily wet-cough, and recurrent oto-sinopulmonary infections. The patient was diagnosed with pneumonia at two days old and admitted to the neonatal intensive care unit (NICU) where he stayed for 14 days on positive pressure without endotracheal intubation. The patient had multiple hospitalizations through his first three years of life due to bronchiolitis and pneumonia and was diagnosed with asthma at four years old. Adenoidectomy and tonsillectomy were done at six years old and myringotomy and three times afterward. Physical examination was pertinent for bilateral nasal polyps, bibasilar crackles, and mild generalized clubbing. Chest imaging showed persistent chronic right middle lobe (RML) and left lower lobe (LLL) atelectasis, and high-resolution computer tomography (HRCT) of the chest showed bilateral cylindrical and varicose bronchiectasis at RML and LLL. Ciliary biopsy was positive for an abnormal number and distribution of microtubules [7+2, 9+0] with central pair defects. Spirometry resulted in forced vital capacity (FVC): 78%, Forced expiratory volume in the first second (FEV1): 71%, FEV1/FVC: 91%, total leucocyte count (TLC): 89%, residual volume (RV): 168%, RV/TLC: 177% percentage of predicted, consistent with a pseudo-restrictive pattern and presence of air trapping. Bronchoscopy showed normal airway anatomy and normal segmental distribution. Abundant thick and greenish secretions bilaterally were observed mostly in the RML and LLL. Bronchoalveolar lavage fluid resulted positive for *Pseudomonas aeruginosa* pulmonary infection. The sweat test was in 32.3 mEq/L on the right arm and 35.6 mEq/L on the left arm. Genetic testing results were negative for cystic fibrosis transmembrane conductance regulator (*CFTR*) genetic mutations. Serum immunoglobulin titers were normal, except for an elevated immunoglobulin E of 902 UI/mL. The genetic panel for primary immunodeficiencies showed a heterozygous variant of unknown significance (VUS) at the *CTC1* gene c.2803C>T (p.Leu935Phe). Fractional exhaled nitric oxide (FeNO) was measured, resulting in <5 ppm. Nasal nitric oxide (nNO) was unavailable. Genetic sequence analysis and deletion/duplication for 36 PCD genes was completed and revealed one pathogenic variant at the *RSPH4A* gene [c.921+3_921+6delAAGT] splice site mutation. Additionally, one likely pathogenic variant was present: *RSPH4A* [c.1103T>G (p.Val368Gly)] and a VUS in *DNAH8* [c.9839A>T (p.Gln3280Leu)]. Familial genetic studies confirmed the maternal inheritance of the *RSPH4A* [c.1103T>G (p.Val368Gly)] variant. No paternal *RSPH4A* or *DNAH8* variants were detected. As a result of the subsequent familial analysis, reclassification was made for the *RSPH4A* [c.1103T>G (p.Val368Gly)] variant as pathogenic.

Case 2: *RSPH4A* homozygous female pediatric case

A six-year-old native Puerto Rican female presented with a past medical history of daily wet cough, recurrent pulmonary infections, bronchiectasis, sinusitis, and neonatal respiratory distress despite term gestation that required NICU admission for 14 days. Multiple hospitalizations were reported through her first six years of life due to chronic bronchitis and recurrent pneumonias. The patient had a prior surgical history of adenoidectomy at four years old. Physical examination revealed bibasilar crackles, monophonic wheezes, and mild generalized clubbing. Chest imaging showed bilateral opacities concerning for pneumonia, and HRCT of the chest showed bilateral cylindrical bronchiectasis on RML, LLL, and lingula. Ciliary biopsy revealed rare cilia but was not diagnostic for PCD. Spirometry was consistent with air trapping with FVC: 107%, FEV1: 102%, FEV1/FVC: 95%, TLC: 132%, RV: 244%, and RV/TLC: 174% percentage of predicted. Bronchoscopy revealed normal airway anatomy and the presence of bilateral whitish bronchial secretions at the distal airways. Bronchoalveolar lavage fluid was negative for pulmonary infection. A follow-up outpatient throat culture was positive for *Pseudomonas aeruginosa*. The sweat test was 15.7 mEq/L on the right arm and 13.2 mEq/L on the left arm. Genetic testing results were negative for *CFTR* and primary immunodeficiencies genes. Serum immunoglobulin titers were normal. The FeNO test was <5 ppm. Measurement of nNO was unavailable. A homozygous pathogenic *RSPH4A* gene [c.921+3_921+6delAAGT] splice site mutation was identified on subsequent PCD genetic testing. Additionally, a pathogenic variant in *DNAH11* [c.3133C>T (p.Arg1045*)] was present. The maternal genetic analysis detected the presence of the *RSPH4A* mutation but the *DNAH11* genetic variant was not detected. Paternal testing was not available.

Methodology and results of the ancestral *RSPH4A* analysis

**Figure 1:**
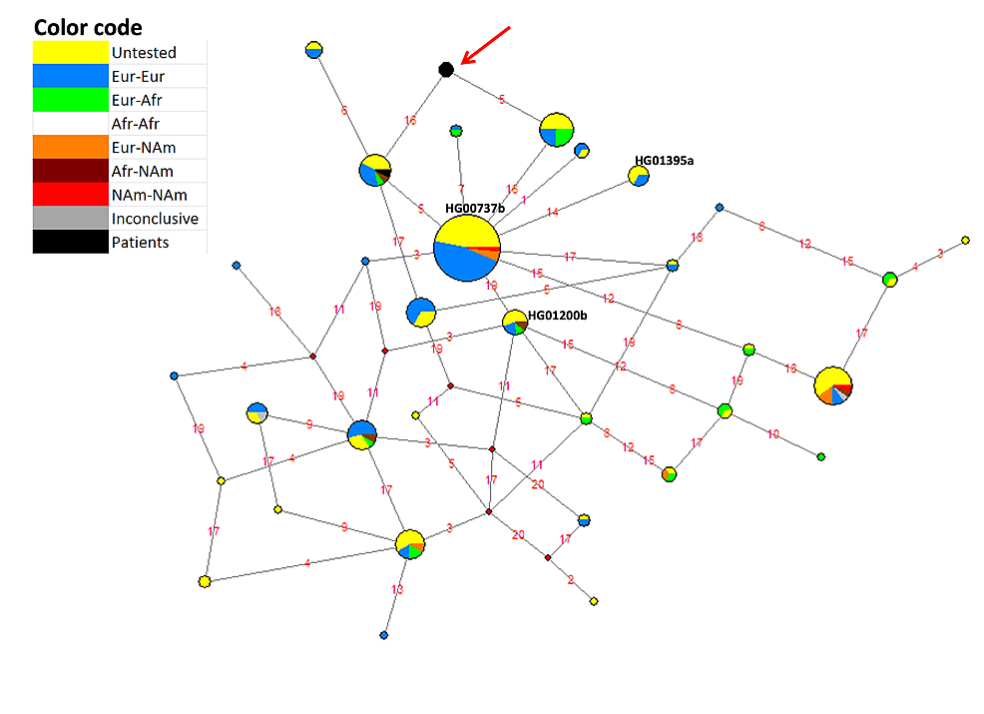
Haplotype network of the region spanning from nucleotide positions 116,864,852 to 117,021,275 in chromosome six in 104 Puerto Rican subjects in the 1000 GP. Haplotypes are represented by circles of size proportional to the number of chromosomes they represent. Hypothetical haplotypes are represented by red diamonds. Haplotype colors refer to their predicted ancestry. A haplotype that represents only patient chromosomes is highlighted with a red arrow. Lines connect haplotypes differing by polymorphisms that are represented by red numbers: 1 = rs2352482, 2 = rs117750891, 3 = rs17078051, 4 = rs76319074, 5 = rs7752566, 6 = rs11755235, 7 = rs62424373, 8 = rs9481650, 9 = rs41289942, 10 = rs111375012, 11 = rs6927567, 12 = rs9488991, 13 = rs146142715, 14 = rs13202520, 15 = rs9488999, 16 = rs72959988, 17 = rs9489008, 18 = rs1165376, 19 = rs9481657, 20 = rs72962017. Black numbers next to three haplotypes indicate the haplotypes to which three chromosome copies with the *RSPH4A* [c.921+3_921+6delAAGT] splice site mutation in the 1000GP belong.

To confirm the *RSPH4A* [c.921+3_921+6delAAGT] splice site mutation ancestry, the Phylogenetic Network Software of fluxus-engineering.com was used to construct a median-joining haplotype network [13] with genome sequence data from 104 Puerto Rican subjects in the 1000 Genomes Project (1000GP) [14]. The haplotype network (Figure 1) was constructed using the 20 polymorphic sites genotyped by 23andme that are located from nucleotide positions 116,864,852 to 117,021,275 in chromosome six, according to the GRCh37 human genome assembly. The span starts 72,790 bp upstream of the transcription initiation site of the *RSPH4A* gene and ends 67,127 bp downstream of its 3’ end.

The most common haplotype in Figure 1 consists only of non-African chromosomes, and likely represents an out-of-Africa founder haplotype from which most of the remaining non-African haplotypes arose. One of the 62 chromosome copies that compose this haplotype (HG00737b) possess the *RSPH4A* [c.921+3_921+6delAAGT] splice site mutation, and two other haplotypes, each differing from the founder haplotype by a single polymorphism, also contain a chromosome copy with the mutation (HG01200b and HG01395a). The 1000GP data estimates the frequency of the *RSPH4A* [c.921+3_921+6delAAGT] splice site mutation in Puerto Rico at 1.4% (3/208). It is a low-frequency but not rare allele in Puerto Rico in spite of its pathogenicity. Furthermore, the *RSPH4A* [c.921+3_921+6delAAGT] splice site mutation seems to have arisen in an out-of-Africa founder haplotype. At least two subsequent mutations, identified in Figure 1 as polymorphisms #14 and #19, generated new haplotypes holding the *RSPH4A* [c.921+3_921+6delAAGT] splice site mutation.

Figure 1 also shows a haplotype that only consists of three patient chromosome copies (red arrow). Two of the three chromosomes belong to a patient homozygous for the *RSPH4A* [c.921+3_921+6delAAGT] splice site mutation (Case 1), and the other to a compound heterozygous patient holding one copy of the same mutation (Case 2). The remaining chromosome of the latter patient differs only at rs72959988 (SNP #16 in Figure 1). Thus, it is highly likely that the three patient chromosomes with the *RSPH4A* [c.921+3_921+6delAAGT] mutation share the same, unique haplotype, which, for simplicity, we will call “the disease haplotype.” In conclusion, the data from our patients strongly suggest that, in addition to polymorphisms #14 and #19, a third polymorphism arose in the founder haplotype, either polymorphism #5 or polymorphism #16, to generate another haplotype holding the *RSPH4A* [c.921+3_921+6delAAGT] splice site mutation. This third derived haplotype with the *RSPH4A* [c.921+3_921+6delAAGT] splice site mutation recombined with a haplotype holding either polymorphism #5 or polymorphism #16 to generate the disease haplotype, which possesses both polymorphisms #5 and #16.

The three mutations occurring after the rise of the *RSPH4A* [c.921+3_921+6delAAGT] splice site mutation allow us to estimate its time of origin. Applying the human nucleotide nuclear mutation rate of 2.5 x 10-8 mutations per nucleotide site to the studied fragment, which is 156,423 bp long, and using 30 years to represent generation time [15], we estimate the age of *RSPH4A* [c.921+3_921+6delAAGT] splice site mutation at 23,015 years. This is approximately 8,000 years prior to human arrival to the New World.

## Discussion

Previous studies have identified the founder mutation *RSPH4A* [c.921+3_6delAAGT] in the Hispanic Puerto Rican population [7,10]. This study explored the ancestry of the founder mutation in two genetically confirmed PCD subjects with genetic mutations in the *RSPH4A* gene. Determining the origin of a mutation can be a difficult task, especially in a highly admixed population like Puerto Rico. In this study, we base our analysis on three lines of evidence: local ancestry of chromosomal segments carrying the *RSPH4A* [c.921+3_921+6delAAGT] splice site mutation, the origin of neighboring haplotypes in the haplotype network, and age.

Phase 1 of the 1000GP presented the genome sequence of 55 Puerto Ricans, all of which were subjected to local ancestry analysis [14]. A female of these 55 Puerto Ricans (HG00737) held the *RSPH4A* [c.921+3_921+6delAAGT] splice site mutation in its heterozygous form, but both of her chromosomal segments holding the *RSPH4A* gene were determined by the local ancestry analysis to be of European origin. It is thus likely that the *RSPH4A* [c.921+3_921+6delAAGT] splice site mutation arrived in Puerto Rico from Europe.

The haplotype of this chromosome segment is a likely founder of the out-of-Africa migration that started populating the rest of the world some 60,000 years before present (YBP). However, we estimate the mutation to have arisen only 23,015 YBP. Hence, the haplotype had plenty of time to spread all over Eurasia before the mutation arose. However, the data suggest that the geographic extension of the mutation is very limited because it only appeared in three Puerto Ricans and not once in any of the other 25 world populations that were sampled by the 1000GP. The mutation is estimated to have arisen before the colonization of the New World from a very small Siberian population approximately 15,000 YBP [16]. Considering the bottleneck effect that occurred during this event, and the fact that the Caribbean was the last region of the Americas to be colonized by humans [17], had the mutation arrived in Puerto Rico from its Native Americans, it would have been reasonable to expect that it must have been present and found in other American populations.

These findings contrast the original hypothesis suggested by Leigh et al., who attributed the origin of the founder mutation to the Taino ancestry due to the presumable prevalence of recessive conditions maternally inherited through mitochondrial genes and favored by the island’s geographically isolation [7,12]. Future research could consider the analysis of this dataset with an additional range of chromosomal segments to further explore the origin of the *RSPH4A* [c.921+3_6delAAGT] splice site mutation, the actual prevalence of the condition on the island, and the applicability of the approaches used to date.

Finally, additional facts supporting the European origin of the founder mutation *RSPH4A* [c.921+3_6delAAGT] are: first, the mutation has a single origin implied from the type of mutation, and the 1000 GP Database identified unambiguously in the HG00737b mutation is from European origin. Hence, the two patient’s chromosomes have a single origin, and one is strictly European. Furthermore, haplotype HG01395a, which has the mutation of interest, only contains chromosomes of European origin.

## Conclusions

One of the conclusions of the 1000GP was that deleterious mutations were commonly limited to single populations. As the age of *RSPH4A* [c.921+3_921+6delAAGT] bars the possibility that it arose in Puerto Rico after its colonization by Europeans, it is likely that its origin arose from a limited region within Spain. The diversity of haplotypes holding this mutation strongly suggests that the region was a big source of colonists who brought such haplotypes to Puerto Rico in early colonial times. The haplotypes quickly increased in frequency due to the genetic drift and the population expansion that followed. As a conclusion, the ancestral haplotype analysis presented shows that chromosome segments with haplotypes equal to those of the chromosomes carrying the *RSPH4A* [c.921+3_6delAAGT] splice site mutation had predominantly European ancestry.
